# Activation of Anthracite Combustion Using Pyrolysis Oil from Thermal Conversion of Waste Car Tires

**DOI:** 10.1021/acsomega.1c02404

**Published:** 2021-07-21

**Authors:** Kirill B. Larionov, Konstantin V. Slyusarskiy, Svyatoslav A. Tsibulskiy, Albert Zh. Kaltaev, Nikolay I. Berezikov, Alexander S. Gorshkov, Sergey V. Lavrinenko, Vladimir E. Gubin

**Affiliations:** † School of Energy & Power Engineering, National Research Tomsk Polytechnic University, Tomsk 634050, Russia; ‡ Laboratory of Catalysis and Processing of Hydrocarbons, National University of Science and Technology “MISIS”, Moscow 119049, Russia

## Abstract

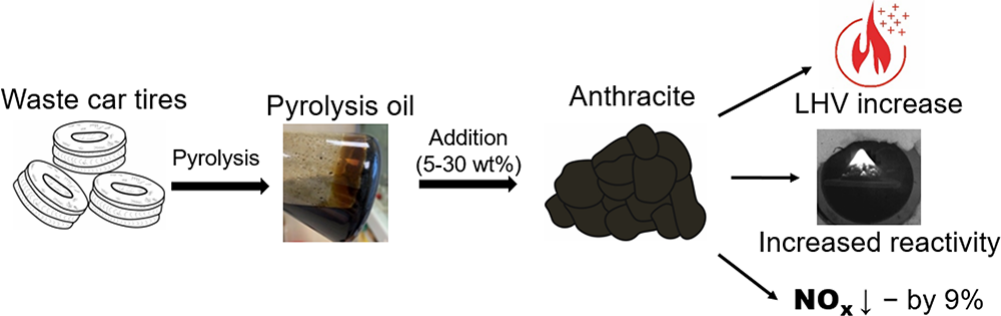

The ignition and combustion of anthracite modified by the addition of pyrolysis oil obtained during thermal processing of waste car tires (WCTs) had been studied. The mass fraction of WCT pyrolysis oil was varied in the range from 5 to 30 wt %. The additive was applied by the drop impregnation method. Ignition and combustion of obtained samples were carried out in a combustion chamber at temperatures of the heating medium *T*_g_ = 600–800 °C. The gas-phase combustion products were analyzed using an in-line gas analyzer. The application of WCT pyrolysis oil as a combustion modifier contributed to an increase in the reactivity of anthracite, which was expressed in a decrease in the minimum ignition temperature (by 23–104 °C) and a reduction in the ignition delay time. The high-speed video recording indicated that the combustion of both initial and modified with 5 wt % pyrolysis oil anthracite samples was realized in oxidation mode. For samples with more than 10 wt % pyrolysis oil additive, the formation of a visible flame was observed near the sample surface. With an increase in the mass fraction of the additive, the rate of combustion front propagation was increased. The application of WCT pyrolysis oil as a combustion modifier also contributed to the reduction or even the almost complete elimination of unburnt carbon content in the ash residue formed after anthracite combustion.

## Introduction

Coal is one of the main fuels for power generation worldwide.^1^ Its consumption declines very slowly making it relevant for the upcoming decades. However, depletion of traditional energy-grade coal requires power plants to adjust to available coal. The most troublesome case is switching to low-reactivity solid fuels like anthracite or coke. In order to ensure reasonable stability of combustion and fuel burnout in a wide range of operational parameters, the significant modification of boilers is required.^2,3^ The other solution is using different additives in order to improve ignition properties of such fuels. The common solution is using traditional catalysts based on Ca,^4,5^ Fe,^4,6^ Cu,^7^ and Ce^8^ in the form of oxides^5,6^ and precursors.^7^ However, despite a small mass of such additives being required, due to high consumption of coal in power plants, the cost of such a solution sometimes could be unacceptable. The cheaper option is blending coal with some highly reactive fuel to promote its ignition. The second component for such a mixture could be brown coal,^9^ biomass^10,11^ and wastes of different types.^12−14^ Recent studies revealed that application of liquid additives^15−18^ allow significant improvement of both ignition and combustion characteristics of initial coal even with a small mass of additive: up to 10 wt % according to ref (15) and up to 5 wt % according to ref (17) (with the optimum value at 3 wt %). Furthermore, it has been theorized that^19^ combined application of pyrolysis oil with coal at power plants will allow significant reduction of the contribution to the greenhouse effect. However, the effect of a very limited number of liquids has been investigated so far.

The common method for studying the effect of different additives on the reactivity of an obtained mixture with coal is thermogravimetric analysis.^6,9,10,14,20^ The promoting effect is assessed via comparison of characteristic parameters like ignition^10^ and burnout temperatures^14^ as well as values of kinetic constants.^9,11,13^ However, conditions in thermogravimetric analyzer chambers differ significantly from those of actual energy equipment in terms of heating rate and oxidizer flow rate pattern. That is why many articles on combustion of different fuels in pilot-scale setups^3,21^ or laboratory units with close to actual equipment conditions^5,7^ have emerged recently. They allow researchers to obtain the most relevant data on expected fuel behavior during actual implementation. However, such works are very scarce and, usually, focus on a chosen aspect of the process like ignition^17^ or flame structure.^15^ Complex research on combustion of coal–pyrolysis oil mixtures has not been performed yet.

Current progress in the research of coal–pyrolysis oil mixtures in general is limited by several studies. Wang et al.^22,23^ performed large research on rheological properties and steam gasification in a lab reactor of fast pyrolysis oil mixed with Yangquan anthracite. The good stability of the obtained slurries and relatively high calorific values of the obtained syngas were reported. The large study on combustion of two coals and char mixed with three different pyrolysis oils was performed by Feng et al.^18^ by means of thermal analysis. The synergetic effect was observed for all blends studied with the most prominent one for low reactivity char, allowing them to conclude that for low reactivity fuel, the pyrolysis oil additive will be more effective. The features of complex water–coal–-pyrolysis oil droplet combustion were studied.^16^ The small additives of pyrolysis oil (up to 8 wt %) to a long-flame coal–water mixture resulted in a moderate (by ∼15 to 20%) decrease in ignition delay time, while 50 wt % additives led to the ignition delay time decreasing by 4 times. Data on combustion behavior of pyrolysis oil–anthracite mixtures were not found.

The current study presents the results of complex research on ignition, combustion, and gas-phase product formation of Siberian anthracite mixed with waste tire pyrolysis oil.

## Results and Discussion

### Ignition

**Figure 1:**
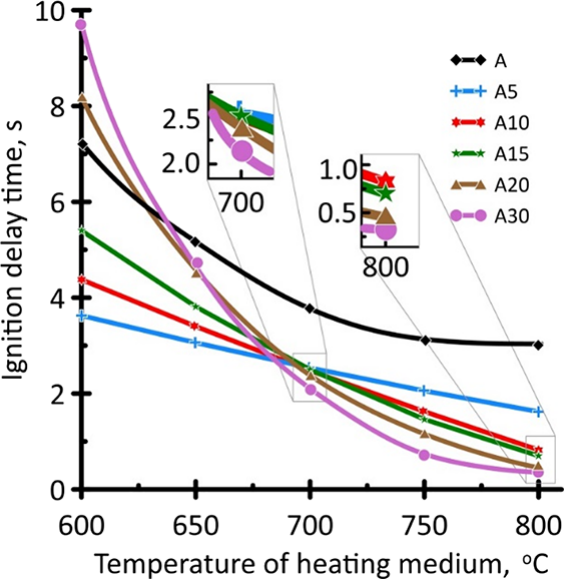
Dependence of the ignition delay time on the temperature of the heating medium (*T*_g_ = 600–800 °C) for samples with different contents of pyrolysis oil.

Figure 1 presents the dependences of the ignition delay time *t*_i_ on the temperature of the heating medium (*T*_g_ = 600–800 °C).

Figure 1 shows that with an increase in the heating medium temperature, an either linear (for samples A5, A10, and A15) or exponential (for samples A, A20, and A30) decrease in the ignition delay time was observed, which was in good agreement with the data of other authors for a coal–water slurry with a waste turbine oil additive.^24^ It is important to note that the addition of 5–15 wt % pyrolysis oil (samples A5, A10, and A15) at *T*_g_ = 600 °C initiated the ignition of anthracite, which was shown by the decrease in *t*_i_ value by 25–50%. However, the different behavior was observed for A20 and A30 samples. For these samples, *t*_i_ was increased by 13 and 35%, respectively. Thus, the following activity row could be formulated: A30 > A20 > A > A15 > A10 > A5. This effect could be associated with a relatively low *T*_g_ temperature, which ensured intense evaporation of water and early sublimation of light hydrocarbons of the pyrolysis oil. Thus, it was assumed that the ignition of samples modified with pyrolysis oil could be characterized by the following stages:

desorption of water,
thermal conversion of volatile compounds of anthracite (Table 1) and combustible substances of pyrolysis oil (Table 2),
release of combustible gas-phase compounds, and
gas-phase ignition.

**Table 1:** Technical Characteristics and Elemental Composition of Anthracite

technical characteristics				elemental composition				
M (wt %)^a^	A (wt %)^d^	VM (wt %)daf	Q (MJ/kg)^a^	C (wt %)^d^	H (wt %)^d^	N (wt %)^d^	S (wt %)^d^	O (wt %)^d^
2.1	17.7	7.2	24.8	69.9	1.4	1.2	0.2	9.6

a Working mass basis,

d Dry mass basis,

daf Dry ash free mass basis.

**Table 2:** Technical Characteristics of the Pyrolysis Oil

parameter	value
density at 15 °C, kg/m^3^	912
kinematic viscosity at 40 °C, mm^2^/s	10.6
dynamic viscosity at 40 °C, mPa·s	9.7
*T*_pp_, °C	–52
*T*_fp_, °C	82
ash content, wt %	n/a
Q^a^, MJ/kg	43.3

Elemental composition, wt%	
C	86.3
H	11.0
N	0.6
S	0.7
O	1.4

fractional composition	
temperature, °C	vol %
98	10
103	20
105	30
107	40
108	50

a Working mass basis.

With an increase in the heating medium temperature (*T*_g_ = 700 °C), almost the same *t*_i_ value was observed for all modified samples, which was ∼2.4 s. At 800 °C, a following activity raw could be formed: A30 > A20 > A15 > A10 > A5 > A. The observed effect could be associated with the intensification of moisture evaporation and sublimation of light hydrocarbons of pyrolysis oil. Thus, a higher content of pyrolysis oil in the sample composition promoted the release of combustible compounds, the concentration of which was sufficient to provide an earlier gas-phase ignition of the studied sample.

In general, the activation of the anthracite ignition by the pyrolysis oil was most likely associated with the phase transformation of liquid hydrocarbons and their contribution to the quicker reaching of concentration threshold of the combustible gases in the vicinity of the sample.

**Figure 2:**
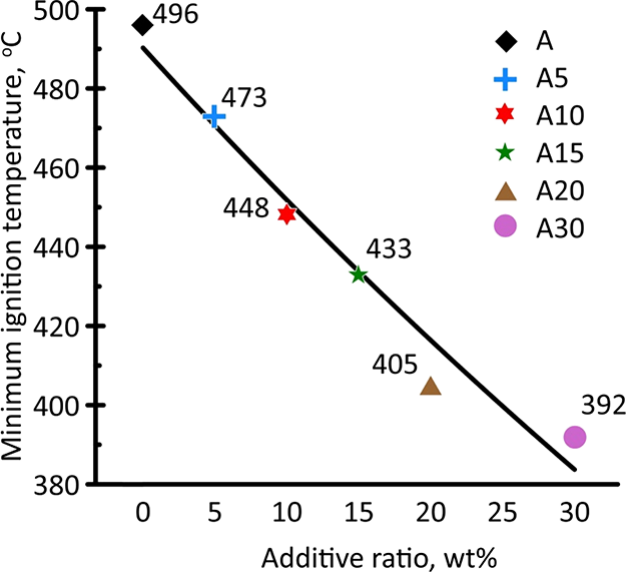
Minimal ignition temperature of studied anthracite samples with different pyrolysis oil content.

Figure 2 is a summary diagram illustrating the variation in the *T*_i_^min^ for the initial and modified with pyrolysis oil anthracite samples.

For sample A, the minimum ignition temperature was 496 °C. The application of pyrolysis oil reduced *T*_i_^min^. Thus, with an increase in the additive content (from 5 to 30 wt %), *T*_i_^min^ decreased from 473 to 392 °C. Figure 2 illustrates that the dependence of *T*_i_^min^ on the content of the pyrolysis oil additive had a linear character. Comparison of the results was obtained for the minimum ignition temperature and ignition delay time (Figure 1), and the correlation was observed only at *T*_g_ = 800 °C. At lower temperatures, the regularities were different. According to Vilyunov theory,^25^ the ignition occurs when the heat release rate due to chemical reactions becomes similar to the heat supplied to the sample from the outside. Thus, assuming that the heat release characteristics change additively with pyrolysis oil concentration in the sample, the heat release rate for the anthracite–oil mixture could be obtained as
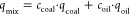
where *c*_coal_/*c*_oil_ is the relative content of coal/oil in the mixture; *q*_coal_/*q*_oil_ is the heat release rate of coal/oil in the mixture.

Considering the heat release rate during thermal conversion of pyrolysis oil at fixed temperature to be higher than for anthracite, the increase of pyrolysis oil concentration will result in a higher heat release rate. Considering the heating conditions of the fuel sample to be the same at different pyrolysis oil concentrations, the linear change in oil concentration will result in a close to linear change at the minimum ignition temperature.

### Combustion

**Figure 3:**
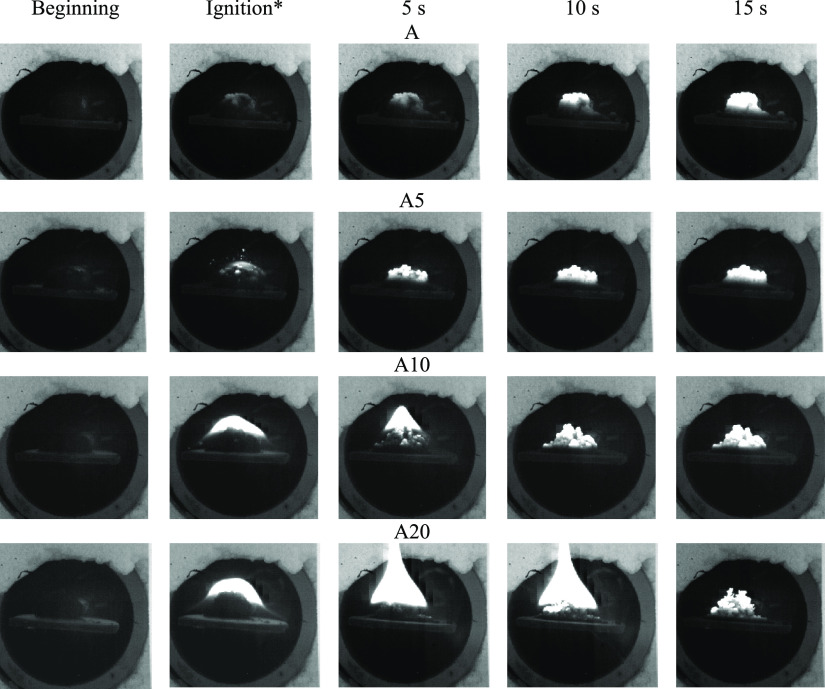
Ignition and subsequent combustion of the studied samples at heating medium temperature *T*_g_ = 800 °C (*ignition delay time: A, 3.1 s; A5, 1.6 s; A10, 0.8 s; A20, 0.5 s).

Figure 3 presents the characteristic frames illustrating the ignition and subsequent combustion of the studied samples at *T*_g_ = 800 °C. Based on the results obtained and published earlier data,^26^ several stages of the physicochemical transformation of fuel could be distinguished: inert heating, moisture evaporation, thermal decomposition of samples and the release of volatile compounds, mixing of combustible gases (including gas-phase products of pyrolysis oil hydrocarbon decomposition) with air, and thermal oxidation of the carbon residue formed.

The propagation of the combustion front for the studied samples had begun after 3 s from the moment of ignition and moved from the surface to the center of the sample fill without a pronounced zone of local ignition. In the time range between 3 and 7 s from ignition, an increase in the glow intensity was observed due to an increase in the temperature of the studied samples.^24^ Flameless combustion (oxidation) of the initial anthracite sample was associated with the low content of volatile matter (Table 1). Thus, during their release, threshold concentrations of combustible gas-phase products (CO, C*_x_*H*_y_*, H_2_) were not reached and the flame was not formed.

For the anthracite samples modified with pyrolysis oil, along with early ignition, a more intense propagation of the combustion front with the formation of a visible flame was observed (for samples with 10–30 wt % pyrolysis oil additive). This may be caused by an increase in the heating rate of the sample during the sublimation of light hydrocarbons of pyrolysis oil and their subsequent oxidation. It could also be assumed that the efficiency of oxygen diffusion in the porous space of anthracite particles was improved due to the appearance of new channels and cracks with intense release and removal of gas-phase products.

**Figure 4:**
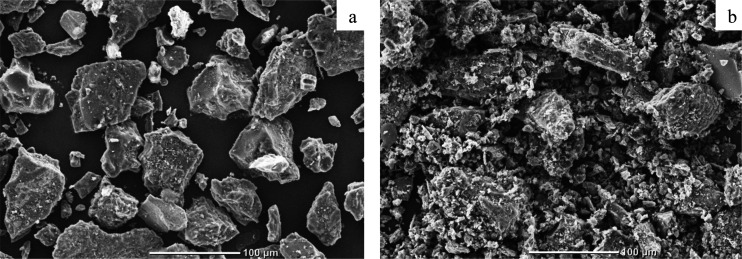
SEM images of partially oxidized samples at heating medium temperature *T*_g_ = 600 °C with ×300 magnitude (a, sample A; b, sample A10).

Figure 4 presents the scanning electron microscopy images of partially oxidized samples A and A10 obtained at 600 °C. The exposure time in the combustion chamber for these samples was 20 s.

The application of pyrolysis oil as an anthracite combustion modifier resulted in the change in the form of the particle surface (Figure 4). It could be seen that the particles of the modified sample (sample A10) had a loose and inhomogeneous surface, which may be associated with the release and subsequent ignition of light combustible compounds and, as a consequence, a faster propagation of the combustion front (Figure 3). It is also worth noting that a smaller ignition delay time for sample A10 (Figure 2) led to the formation of agglomerates consisting of smaller fragments (with <5 μm size) connected to larger particles of anthracite (with >50 μm size).

**Figure 5:**
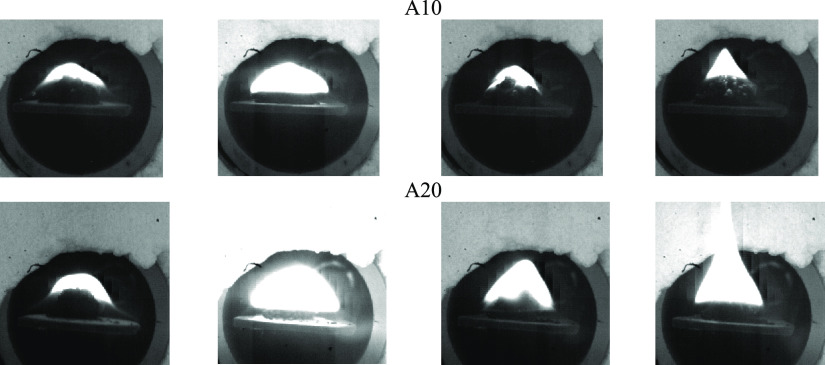
Visualization of the process of gas-phase ignition and subsequent flame combustion of samples A10 and A20 at heating medium temperature of *T*_g_ = 800 °C.

As mentioned earlier, for the modified samples the formation of a visible flame was observed. Its duration depended on the heating medium temperature and the amount of pyrolysis oil added. In this case, the formation of a visible flame was observed starting from 700 °C for samples with less than 10 wt % additive. With an increase in the temperature of the heating medium up to 800 °C, the duration of flame combustion was increased. It also was in good agreement with the results of other authors for combustion of a coal–water slurry mixed with waste turbine oil additives.^27^ The formation of a flame was associated with the intense evaporation of light hydrocarbons of the pyrolysis oil and the simultaneous release of volatile matter of anthracite. Later, a vapor cloud of combustible components mixed with air was formed near the sample surface, due to which the gas-phase ignition of the fuel sample occurred (Figure 5).

Following the gas-phase ignition, a gradually developing flame was observed in the vicinity of the fuel fill (for samples A10 and A20) with a pronounced change in the form of its surface, which also agreed with the data presented in Figure 4. This may indicate that the evaporation of pyrolysis oil light hydrocarbons occurred both from the surface and the bulk of the sample.

At an earlier stage of flame combustion (up to 2 s), an intense expansion of the combustion front was observed, the size of which exceeded the initial characteristic size of the fuel sample by several times (Figure 5). Thus, the burning out of the combustible mixture near the surface of the fuel fill contributed to its additional heating. In this case, the formed flame has a fairly stable shape over the entire duration of flame combustion. After the burnout of light fuel components, a noticeable narrowing of the flame combustion front occurred. For sample A10, this narrowing had started at 6 s from the moment of sample introduction, while for sample A20, this was at 12 s. The energy released during gas-phase combustion was sufficient to accelerate the evaporation of the remaining hydrocarbons from the fuel bulk, to promote the propagation of the heterogeneous combustion front from the surface of the fill to its center. This process lasted until the resulting carbon residue was burned out.

It is worth mentioning that for sample A20, during its flame combustion, the formation of a fibrous structure (soot) on the surface of the fuel was observed. Then, the soot formed continued to burn in a heterogeneous mode.

**Figure 6:**
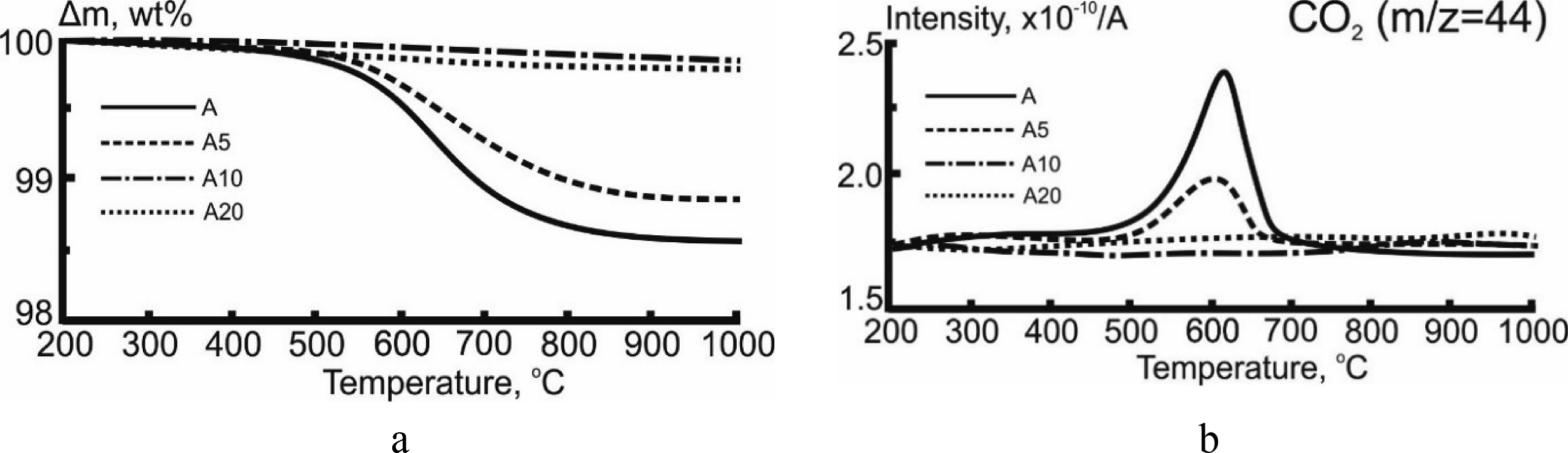
TG (a) and MS (b) results for the air oxidation of the ash residue formed after combustion of samples at heating medium temperature *T*_g_ = 600 °C. Atmosphere, air (150 mL/min); heating rate, 10 °C/min.

Figure 6 presents the thermal analysis results for the oxidation of the ash residue formed after combustion of the studied samples at different temperatures of the heating medium.

According to TG data (Figure 6a), in the temperature range 550–1000 °C, the weight loss was 1.6 and 1.2 wt % for samples A and A5, respectively. This weight loss was associated with the conversion of the unburnt fuel (carbon residue) of the ash formed. The presence of unburnt fuel was also confirmed by MS analysis data (Figure 6b). For samples A and A5, the CO_2_ release rate curve (according to mass charge number *m*/*z* = 44) had a monomodal profile, which was obtained in the temperature range 520–700 °C. When pyrolysis oil was added, a decrease in the intensity of carbon dioxide formation was observed. For samples A10 and A20, the unburnt fuel was not observed. This trend could be associated with the development of the surface structure and porosity of fuel particles (Figure 4) due to the intense release of gas-phase products formed during thermal transformation of anthracite samples with the pyrolysis oil additive.

### Gas-Phase Combustion Products

**Figure 7:**
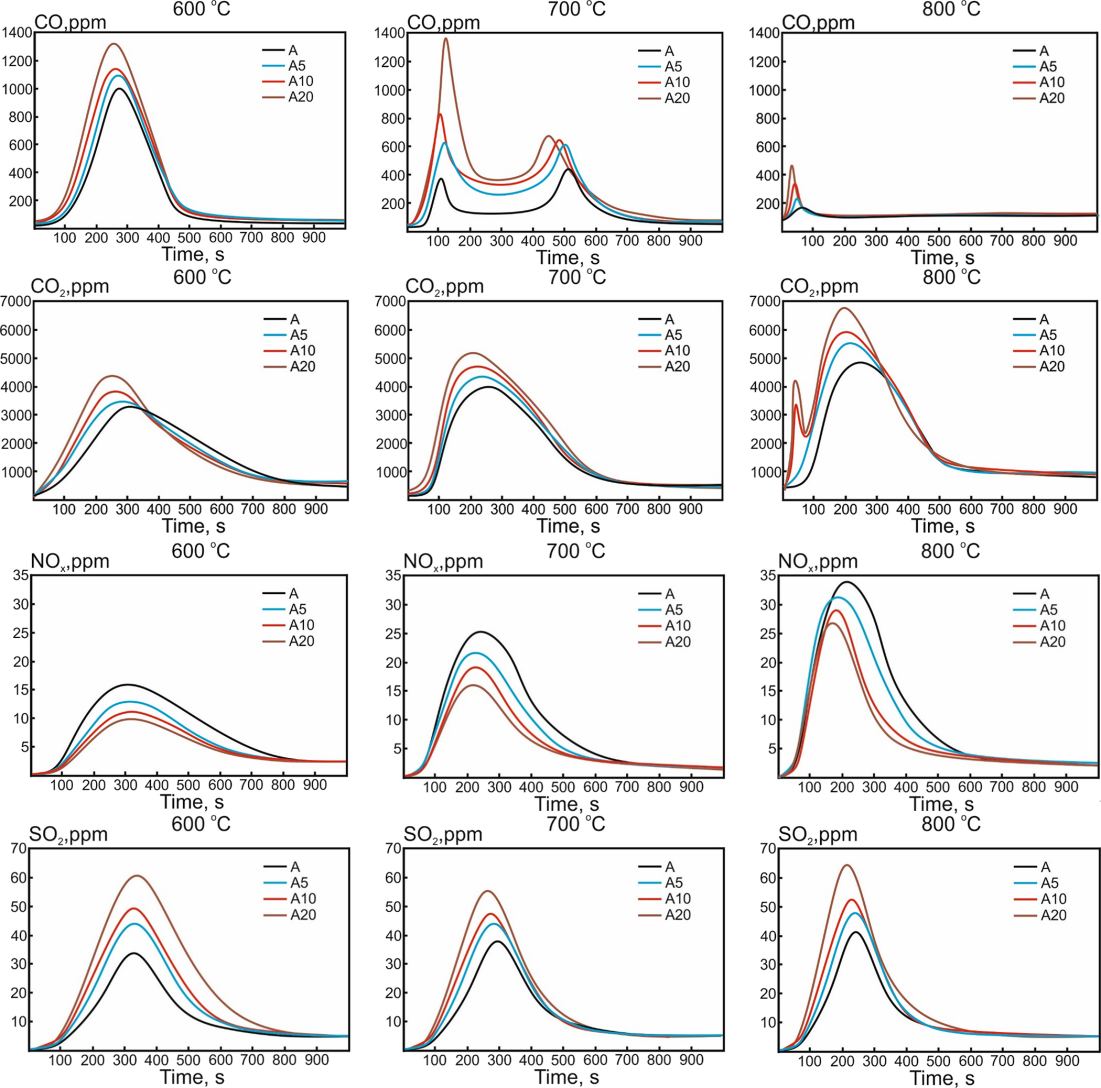
Concentration profiles of the CO, CO_2_, NO*_x_*, and SO_2_ release at different temperatures of heating medium *T*_g_.

Figure 7 illustrates the results of the analysis on the composition of gas-phase combustion products released during the burning of studied samples at different temperatures of the heating medium *T*_g_.

Figure 7 shows that with an increase in the heating medium temperature, the decrease in the concentration of CO in the composition of gas-phase combustion products with a change in characteristic profiles was observed. The profile transformation could be explained by a more intense release of volatile compounds with their subsequent oxidation. In this case, for the modified samples, more intense CO release for longer times was observed due to an increase in the carbon content in the composition of the studied samples (Tables 1 and 2). It is also worth noting that at *T*_g_ = 600 °C, the curves of CO release become bimodal, which was associated with the separation of the stages of volatile compound release, evaporation of light hydrocarbons (for modified samples), and oxidation of the carbon residue. In this case, the release of volatile compounds could also have a staging character, which was characterized by the outflow of volatile substances from the surface and internal volume of the particles of the studied anthracite. In this case, the extremum of the CO release concentration curve was observed at the early stage of coal combustion, where an intense release of volatile substances occurred.

According to Figure 7, the CO_2_ release profiles behaved oppositely to the CO curve. An increase in the maximum concentration of CO_2_ with increase in heating medium temperature *T*_g_ was likely associated with more intense oxidation of CO and the reduction in the total time of fuel combustion. The dependences of the maximum concentrations of CO and CO_2_ on the heating medium temperature were almost linear.

According to ref (28), the formation of NO*_x_* due to oxidation of fuel nitrogen was the dominant mechanism for the studied samples. The decrease in the total nitrogen content in the modified samples (Tables 1 and 2) was the one of the main reasons for the decrease in NO*_x_* concentration maxima with the content of pyrolysis oil additive (Figure 7). With an increase in the heating medium temperature, an increase in the concentration maxima of the NO*_x_* released was observed due to both the involvement of molecular N_2_ from air in the interactions with oxygen and quicker release of fuel nitrogen, which was in agreement with the results of other authors.^28^ In this case, the profiles characterizing the release of SO*_x_* at different heating medium temperatures had a similar character (Figure 7). The greater value of the concentration maxima (expressed in %) for the modified samples could be explained by the increase in the sulfur content in the composition of the obtained samples (Tables 1 and 2).

The similar gas emission features were reported for mixtures of coal with water and waste turbine oil,^28,29^ which indirectly proves correctness of the obtained results.

## Conclusions

In the current article, the results of our experimental study on the ignition and combustion of anthracite modified with a waste car tires pyrolysis oil additive in an amount of 5–30 wt % were presented. The study on the combustion was carried out using an experimental setup for burning solid fuel with high-speed video recording. The addition of pyrolysis oil to the anthracite resulted in an increase in the fuel reactivity, which was expressed in the decrease in the minimum ignition temperature by 23–104 °C and a reduction in the ignition delay time by ∼75% (at *T*_g_ = 800 °C).

Using high-speed video recording, it was found that for the initial and modified with 5 wt % of pyrolysis oil anthracite samples, the combustion was realized in a flameless mode, which was caused by their slow oxidation. For samples with a pyrolysis oil content more than 10 wt %, a more intense propagation of the combustion front was observed with the formation of a visible flame at an early stage of the process. This effect could be associated with the release of a larger amount of combustible gas-phase products formed during the thermal transformation of light hydrocarbons of pyrolysis oil.

The application of the additive also allowed us to reduce the content of unburnt fuel in the ash residue. This could be explained by the active development of the porosity and surface of fuel particles due to the intense release of gas-phase products during combustion, which promoted the diffusion of the oxidizer to the fuel sample.

Quantitative analysis of gas-phase combustion products illustrated that the application of the additive led to an increase in the concentration of the released CO, CO_2_, and SO_2_ (on average by 28, 19, and 11%, respectively). It was associated with an increase in the carbon content in the modified samples. In turn, the concentration of formed NO*_x_* was lower (on average by 9%).

Analysis of the technical characteristics of anthracite and pyrolysis oil allowed us to conclude that the application of the latter as a combustion modifier will contribute to the proportional increase in the net calorific value and decrease in the ash content of solid fuel. Due to the low viscosity and pour point of WCT pyrolysis oil, its storage and application at energy objects are possible even in cold climate conditions. Involvement of the liquid-phase product of WCT pyrolysis into the energy sector will promote a decrease of its carbon footprint and development of a pyrolysis production market.

While both minimal ignition temperature and ignition delay times at high heating medium temperatures were found to nonlinearly and unambiguously decrease with mass of the WCT pyrolysis oil additive, at lower temperatures, the 5 wt % additive resulted in the smallest ignition delay time and increasing the additive mass caused an increase in this parameter. Thus, this method could be used for smooth adjustment of ignition and combustion parameters for application at high temperatures of heating medium.

## Experimental Section

### Materials

Anthracite obtained from the Krasnogorsk deposit (Kemerovo region, Russian Federation) was used as an initial solid fuel sample. Preliminarily, a sample of anthracite (with a fraction size *d* = 5–10 mm) was ground in a drum mill for 8 h with a ratio of mass of grinding bodies to material of 1: 1. Next, the resulting powder was fractionated on sieves with a mesh size of less than 80 μm. Before analytical investigation, the anthracite sample was kept in a lab draft hood for 24 h at 23 °C in order to remove external moisture.

Proximate analysis (moisture M, ash A, volatile matter VM content, and net calorific value Q) was performed according to standard ISO methods: ISO 589:2008, ISO 1171:2010, ISO 562:2010, and ISO 1928:2009, respectively. The mass fraction of the main elements (C, H, N, S, O) in the composition of the investigated sample of anthracite was determined using an elemental composition analyzer Euro EA 3000 (EuroVector, Italy). Before elemental analysis, the sample was dried in an oven at 105 °C until reaching a constant mass. The results of the analysis made are presented in Table 1. Such values are characteristic for this fuel.^30^

As an additive to anthracite, pyrolysis oil was used, obtained by condensation of a vapor–gas mixture formed during pyrolysis of waste car tires (WCT). A sample of pyrolysis oil was obtained from the industrial enterprise NPO Innovatech (St. Petersburg, Russia) in a continuous-type pyrolizer with 500 kg/h capacity at 500 °C. The density of samples at 15 °C and their kinematic and dynamic viscosities at 40 °C were determined via a Stanbinger SVM3000 viscometer (Anton Paar, Austria), according to ISO 12185:1996 and ISO 3104:1994, respectively. The pour point (*T*_pp_) was determined via a CRYO-T-05-01 liquid low-temperature thermostat (Termex, Russia), according to ASTM D97-17b. The flash point (*T*_fp_) was determined in an open crucible using PE-TVO apparatus (Ekros, Russia) according to ISO 2592:2000. The ash content of the studied samples was determined according to ISO 6245:2001 using a muffle furnace. The net calorific value of the studied samples was determined using a ABK-1 bomb calorimeter (Russian Energy Technologies, Russia). Elemental analysis was determined using a Flash 2000 CHNS analyzer (Thermo Fisher Scientific, USA). The fractional composition was determined using ARNS-1E oil distillation apparatus (Neftekhimavtomatika, Russia). The abovementioned characteristics of the pyrolysis oil are presented in Table 2.

According to Table 2, the studied oil sample obtained by WCT pyrolysis was characterized by a high net calorific value, large content of light hydrocarbons, low viscosity, and pour and flash points, as well as the almost complete absence of ash residues. From the energy perspective, this oil could be used in a cold climate without additional heating. Due to the absence of ash residues and a higher net calorific value of pyrolysis oil compared to the studied sample of anthracite (Table 1), their mixing will contribute to a proportional increase in the heat of combustion of fuels and a decrease in ash content. However, the disadvantage of using pyrolysis oil as an anthracite combustion modifier is an increase in the sulfur content (Tables 1 and 2). This disadvantage is partially offset by the lower nitrogen content in the pyrolysis oil, which promotes the reduction in the NO*_x_* emissions from the oxidation of fuel nitrogen. The capture of additional SO_2_ formed during combustion of anthracite mixed with WCT pyrolysis oil could require using the technology of flue gas desulfurization by sorbents.^31^

5, 10, 15, 20, and 30 wt % pyrolysis oil additives were introduced into the anthracite sample by mechanical mixing into a pounder. The mixing time for each sample was ∼5 min. Based on the mass content of the additive, the studied samples will be further referred to as A, A5, A10, A15, A20, and A30. It is worth mentioning that the A5, A10, and A15 samples were powder-like (without large aggregate formation) while A20 and A30 samples had a dispersed (paste-like) form.

### Ignition and Combustion of Studied Samples

**Figure 8:**
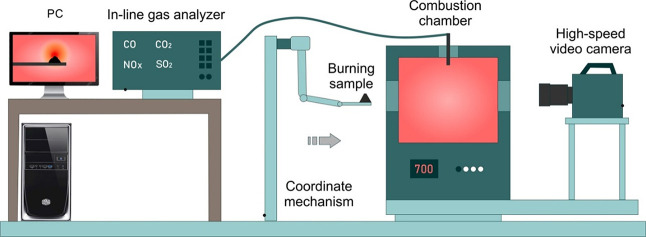
Experimental setup for studying ignition and combustion of solid fuels.

The study of ignition and combustion of studied samples was carried out using an experimental setup, the scheme of which is shown in Figure 8.

The experimental stand (Figure 8) includes the following elements: a combustion chamber in the form of a temperature-controlled PM-1400 (Rusuniversal, Russia) furnace with a digital temperature controller (measurement error ± 1–3 °C) with a volume of 0.012 m^3^; a platform of the coordinate mechanism, which is designed to move the fuel fill into the center of the furnace with an error ± 1 mm; in-line gas analyzer Test 1 (BONER, Russia) for measuring the yield of gas-phase combustion products released (CO, CO_2_, SO_2_, and NO*_x_*); high-speed video camera FASTCAM CA4 5 (Photron, USA) with an image format of 1024p and frequency of 105 Hz.

The weight of the samples studied was ∼0.1 ± 0.01 g. The sample was placed in a cylindrical form mounted on a metal plate. Then, the form was removed by a translational upward movement; as a result, a cone-shaped filling was formed. The required temperature of the heating medium in furnace was set (in the range 600–800 °C, with an intermediate step of 50 °C). It was registered by a chromel–alumel thermocouple. The fuel fill was placed on the holder of the coordinate mechanism, the stroke of which was calibrated along a given coordinate of the combustion chamber center and was controlled via a PC. Simultaneous with the beginning of the rod movement, the video recording has been started. The composition of released gases (CO, CO_2_, NO*_x_*, and SO_2_) was recorded using an in-line gas analyzer.

A comparative assessment of the characteristics of the ignition at different temperatures was performed by analyzing the ignition delay and flame combustion times, determined via a high-speed video camera. The ignition delay time was obtained as the time from the moment of the holder entering the combustion chamber to the moment of the visible glow appearance on the surface of sample, which corresponded to the beginning of the combustion process. The flame combustion time was obtained as the difference between moments of flame formation and extinction. Burning time was considered from the moment of visible glow appearance to the sample burnout with disappearance of the visible flame.

### Minimal Ignition Temperature Determination

The minimum ignition temperature *T*_i_^min^ for the studied samples was determined using the combustion chamber of the experimental stand (Figure 8). The temperature range of *T*_i_^min^ was determined using the bisection method. The required temperature was set in the combustion chamber, and the fuel fill was introduced through the technological hole via a coordinate mechanism. If a visible glow appeared on the surface and/or in the bulk of the fuel fill after 2 min exposure, the ignition was assumed to occur. Then, the temperature of the heating medium was decreased and the procedure was repeated. The minimal temperature of heating medium at which the ignition occurred was considered to be *T*_i_^min^.

### Unburnt Fuel Content in Ash Residue Determination

The unburnt fuel content in the ash residue was determined using a Netzsch STA 449 F3 Jupiter differential thermal analyzer (Netzsch, Germany). The analysis was carried out at a 10 °C/min heating rate in a corundum crucible with a perforated lid upon heating to 1000 °C. ∼20 mg of powder sample was evenly distributed over the bottom of the crucible and placed in a stream of oxidizing medium. The gas flow rate set was 150 mL/min. All experiments were carried out at atmospheric pressure. The qualitative analysis of CO_2_ emission was carried out (for *m*/*z* = 44) using a QMS 403 D Aeolos coupled quadrupole mass spectrometer (Netzsch, Germany).

## Abbreviations

A: ash content, wt %
M: moisture content, wt %
*T*_fp_: flash point, °C
*T*_g_: heating medium temperature, °C
*T*_i_^min^: minimal ignition temperature, °C
*T*_pp_: pour point, °C
Q: net calorific value, MJ/kg
VM: volatile matter content, wt %
WCT: waste car tire
